# A system dynamics approach to understanding the One Health concept

**DOI:** 10.1371/journal.pone.0184430

**Published:** 2017-09-06

**Authors:** Tai Xie, Wenbao Liu, Benjamin D. Anderson, Xiaorong Liu, Gregory C. Gray

**Affiliations:** 1 Faculty of Health Service, Second Military Medical University, Shanghai, China; 2 Faculty of Naval Medicine, Second Military Medical University, Shanghai, China; 3 Division of Infectious Diseases and Duke Global Health Institute, Duke University, Durham, North Carolina, United States of America; University of Rochester, UNITED STATES

## Abstract

There have been many terms used to describe the One Health concept, including movement, strategy, framework, agenda, approach, among others. However, the inter-relationships of the disciplines engaged in the One Health concept have not been well described. To identify and better elucidate the internal feedback mechanisms of One Health, we employed a system dynamics approach. First, a systematic literature review was conducted via searches in PubMed, Web of Knowledge, and ProQuest with the search terms: ‘One Health’ and (concept* or approach*). In addition, we used the HistCite^®^ tool to add significant articles on One Health to the library. Then, of the 2368 articles identified, 19 were selected for evaluating the inter-relationships of disciplines engaged in One Health. Herein, we report a visually rich, theoretical model regarding interactions of various disciplines and complex problem descriptors engaged in One Health problem solving. This report provides a conceptual framework for future descriptions of the interdisciplinary engagements involved in One Health.

## Introduction

The One Health concept is not new [1, 2] perhaps dating back to the late nineteenth century when medical observations were made that human and animal health were closely linked [3, 4]. Today the One Health concept has often been cited an effective approach to complex public health problems that involve multiple disciplines. Often human health, animal health and environmental health are examined as closely linked. Examples of such complex problems include emerging infectious diseases, food safety, and selection of antimicrobial resistant pathogens. In particular, emerging zoonotic disease problems are now remarkable common [5] and very complex. Controlling these complex problems often requires interdisciplinary approaches such as *One Health* [6, 7]. Despite the One Health concept’s growing popularity and acceptance by the professional community, the definition of the term remains imprecise. For example, One Health has been variously defined as an initiative [8], a movement [9], a strategy [10], a framework [11], an agenda [12, 13], an approach, a collaborative effort, among others. As a milestone in the history of One Health, the symbolic ‘umbrella’, developed by One Health Sweden in cooperation with the One Health Initiative Autonomous Pro Bono Team, illustrates the broad One Health scope. According to the ‘umbrella’, the goal of One Heath is to promote a worldwide strategy for the well-being in all aspects of health of people, animals and the environment [2, 14]. As this definition is broad, many disciplines might contribute One Health efforts [15]. Yet, the internal relationships of these disciplines remain a bit amorphous. To better understanding the One Health concept, we sought to review the literature and study key One Health publications with a *system dynamics approach*, providing a systematic representation of the numerous components and how they interact.

## Materials and methods

### System dynamics

*System dynamics* (SD) is a methodology first developed by J.W. Forrester [16] that uses visual and mathematical modeling to simulate and understand complex system problems. Systems thinking is the notion that the system must have structure and the structure determines the system’s patterns of behavior. As such, the patterns of behavior will lead to the system’s output. Therefore, an understanding of system structure helps to better deliver the desired results. Or alternatively stated, thoroughly understanding a complex problem involves discovering the system’s structure, which often involves feedback connections between interdependent components. *Qualitative system dynamics* is used to explain the system’s internal feedback loops to make its relationships easier to understand. This approach has also been successfully used to enhance the development of health policies and programs [17, 18], enable modeling of health care systems [19], and guide chronic disease prevention and interventions [20–22].

A system dynamics theoretical model can be constructed using the following series of steps:

Step 1 **Problem Identification.** Make clear the problem you want to study.
Step 2 **Behavior Analysis.** Carefully identify the elements or components of the system. Analyze the influence of the most crucial behavioral factors involved in the system, either positive or negative, and attempt to explain the cause of the identified problem.
Step 3 **Model Hypothesis.** Propose a hypothesis explaining the results of the second step, and illustrate the model hypothesis in the form of a causal loop, showing the clear mathematical feedback relationships.
Step 4 **Model Evaluation.** Evaluate the model and make sure that it reflects the behaviors and relationships presenting in the system. If it does not, revisit the steps above, else proceed to step 5.
Step 5 **Conclusion and Discussion.** Draw conclusions from the system dynamics model.

### Systematic literature search for One Health articles

To identify scientific articles pertaining to the One Health concept, we performed searches in multiple abstract databases, including PubMed, Web of Science, and ProQuest, in February and March 2017. In PubMed the query string used was ‘("one health") AND (concept* OR approach*)’. In Web of Science, it was ‘TS = ("one health") AND TS = ((concept*) OR approach*)’ with the language restriction—English-language only. In ProQuest, we used ‘all("one health") AND all(concept* OR approach*)’ with the language restriction—English-language only and exclusion source types from newspapers and social media reports. All references were extracted into a reference manager—EndNote (version X7.4, Bld 8818) and screened independently by two researchers (TX and WL). This systematic review conforms to the PRISMA statement (see S1 File). Articles were included if they were peer-reviewed and met four additional criteria: 1) addressed the concept of ‘One Health’, not one health care plan, one health center, or other unrelated topics described by the words “one health”; 2) focused on the concept of One Health overall, not just briefly mentioned the term or only covered a particular practice of One Health; 3) were in the form of an article or review; 4) were written in English. In full test screening, the exclusion criteria were: 1) One Health practice in a particular disease or district; 2) One Health concept or approach mentioned in limited sentences.

To ensure that we did not omit important literature using the aforementioned search strategy, we also used the HistCite^®^ tool to identify significant articles in One Health. HistCite^®^ is a science tool that visualizes the citation relationships between papers to identify key publications in the results returned by a Web of Science search. We used the query string ‘One Health’. Articles were ranked by each publication's Local Citation Score (LCS), which describes how many times it has been cited by the other publications returned by the search. The results were analyzed with HistCite^®^ software version 12.3.17 (Thomson Reuters, New York City, NY, USA). The default setting for searched articles in the HistCite^®^ software is 30 but we increased this default number to 100 articles.

## Results

**Fig 1:**
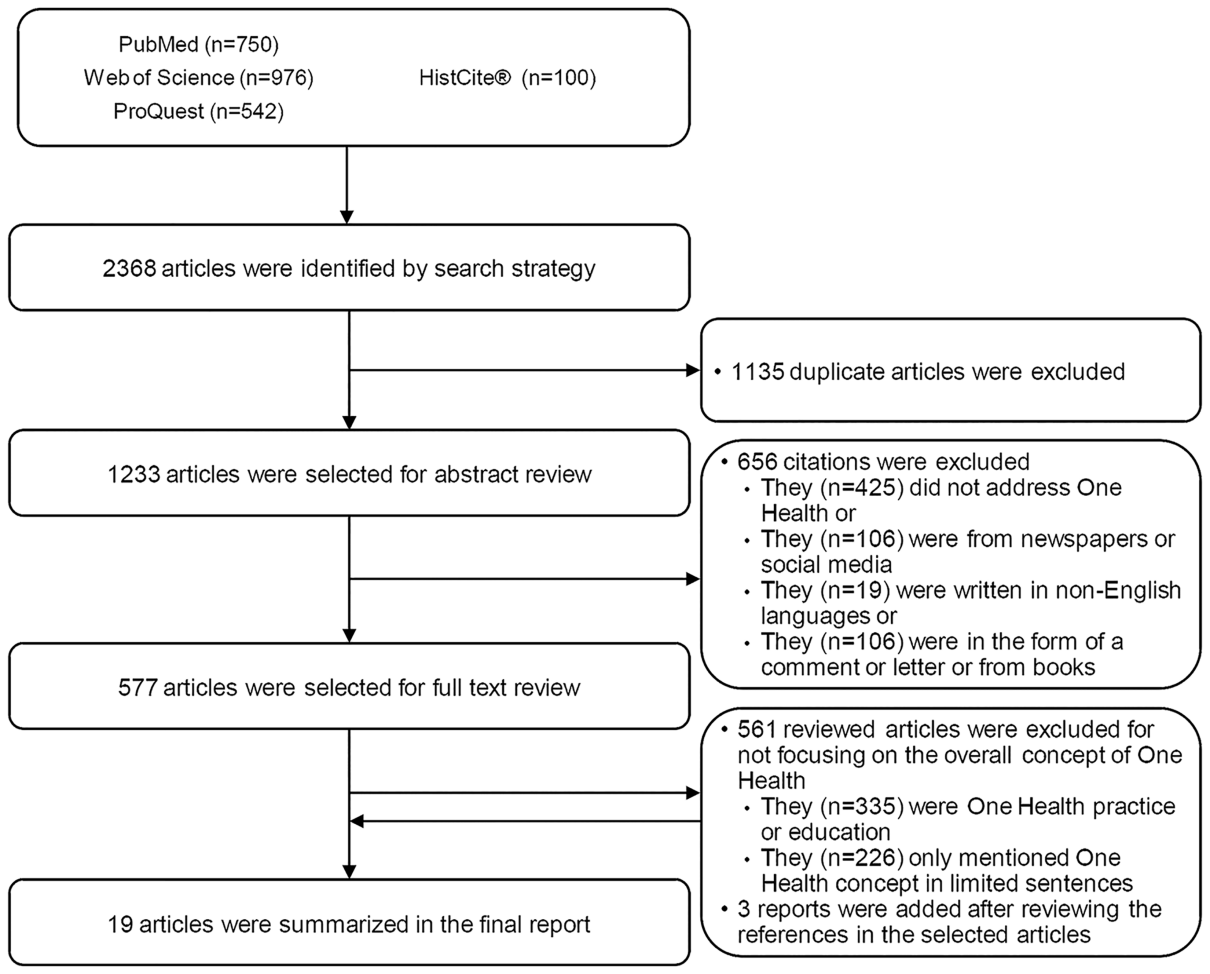
Flow diagram of the literature search process. Using the search strategy, a total of 2368 articles were identified. Duplicate articles were removed. Nineteen publications were used in the final analyses. See text for more details.

The initial search identified 2368 articles: 750 from PubMed, 976 from Web of Science, 542 form ProQuest, and 100 from the HistCite^®^ analysis (Fig 1). One thousand and one hundred thirty-five duplicate reports were manually excluded using reference management software. The abstracts of the remaining 1233 articles were reviewed. Of these, 550 articles were excluded for not addressing the concept of One Health, for originating from newspapers or social media platforms, or for being written in a language other than English. An addition, 106 articles were excluded for being comments or letters or from books. The full texts of the remaining 577 articles were reviewed, after which 561 reports were excluded for lacking a primary focus on One Health or the One Health concept. Three additional report were added during the full text review of articles. In total, 19 remaining publications were included in the subsequent analyses (Table 1).

**Table 1:** Nineteen key One Health articles selected for component analyses.

Publications	Components
Cardiff et al, 2008 [24]	pathology, comparative pathology, genomic biology, food safety, emerging infectious disease
Frank, 2008[25]	veterinary medicine, food security, food safety, emerging infectious diseases, ecosystem protection, comparative medical, human physical/mental health, public health, human medicine, bio-engineering, animal science, environmental science, wildlife
Kahn et al, 2008[4]	veterinary medicine, food supply, intensive agricultural practices, exotic animals global trade, consumption of “bush meat”, human population pressures, comparative medicine
Gibbs et al, 2009[9]	emerging disease, medicine and veterinary medicine, wildlife, public health, education, cross-species disease transmission, media publications
Monath et al, 2010[10]	zoonotic agents, veterinary medical, laboratory animal research, public health, environmental health, biomedical,research, clinical medicine and surgery, biomedical research, ecosystem management, public health, food and agricultural systems safety, biosecurity
Coker et al, 2011[11]	public health, economic security, social stability, health service organisation, patterns and provision and access, fiscal systems, regulation and governance, information systems
Zinsstag et al, 2011[26]	veterinary medicine, public health, animals and wildlife
Rabozzi et al, 2012[27]	public health, clinicians, public health workers, veterinary medicine and veterinary public health officials
Zinsstag et al, 2012[28]	zoonotic infections, livestock and wildlife, global public health, livestock production and wildlife conservation
Calistri et al, 2013[29]	infectious diseases, globalization, animal products trade, wild animals, bush meat, zoonoses agents, public health policy, food-agricultura, veterinary medicine
Atlas, 2013[3]	infectious diseases, vaccination, epidemiology, medical education and clinical care, global travel and commerce, medical education and clinical care
Gibbs, 2013[7]	public health, biomedical research, global food safety and security, ecosystem health, caring for animals, public health, food safety, epidemiology, population medicine, foreign animal diseases
Bidaisee et al, 2014[30]	zoonoses, food safety, agriculture, infectious diseases, livestock into wildlife, scientific and policy challenges, the social, cultural, political norms, veterinary medicine, public health, clinical practice
Evans et al, 2014[6]	epidemiological globalisation, pathogen adaptation, changing human demographics, evolving animal production systems, climate change, water, pollution and environmental contaminants, food safety, food sufficiency and insecurity, the universal global condition of rapid environmental change, new drugs, biodiversity, epidemics, pests, food security, economic prosperity
Gibbs, 2014[2]	veterinary profession, medical profession, wildlife specialists, environmentalists, health policy analysts, social scientists, humanities scholars
Stephen et al, 2014[31]	infectious disease, veterinary, medical and environment sectors, wildlife, animal welfare, food safety and security, drugs and vaccines, public health, training and research
Lerner et al, 2015[15]	infection biology, contagious diseases, zoonotic infections, evolutionary medicine, comparative medicine, translational medicine, biology, human medicine, veterinary medicine, public health, environmental chemistry, health economy, zoonotic diseases, epidemics and toxicants, food-producing animals, pet ownership, education
Stadtlander, 2015[32]	medicine, veterinary medicine, microbiology, public health, biogeography, ecology, environmental, biology
Kingsley, 2016[33]	parasitologists, biosecurity, infection control risks, zoonotic infections, human and veterinary medicine, food security, healthy diets, climate change

### Problem Identification (Step 1)

From the articles identified by the literature review, it was difficult to determine a generally accepted definition for the One Health concept, including the scope and breadth of its practice. This lack of consensus supported the value of our system dynamics theoretical model in developing a conceptual framework to better understand One Health. This concept of One Health served as the root or first level of the theoretical model.

### Behavior Analysis (Step 2)

All 19 analyzed reports agreed that the One Health concept contained at least three primary domains—human, animal, and environment/ecosystem. These three domains were added as the second level of the theoretical model. However, the articles markedly differed regarding their description of the scope of One Health. This discrepancy is likely due to the ongoing rapid development of One Health and the addition of new disciplines that are embracing it as a way forward [23]. Table 1 summarizes the various and diverse disciplines or scopes mentioned in each of the 19 publications, which highlights the broad interpretation that could result in defining the One Health concept. These disciplines and scopes were included as the third level of the theoretical model.

### Model Hypothesis (Step 3)

In the theoretical model, the direction of influence is indicated by the polarity of a causal loop, represented by connecting arrows, which may be positive (“+”) or negative (“-”). A positive causal link indicates that both the causative and the resultant factors increase or decrease in the same direction. A negative causal link indicates that the two linked factors change in opposite directions. Additionally, should two or more elements in the model have a reinforcing or non-reinforcing relationship, the model can be annotated such that these associations can be visualized. This was done by placing a “R” within a clockwise cycling arrow between elements that did have a reinforcing relationship, and a “B” for those elements that did not.

**Fig 2:**
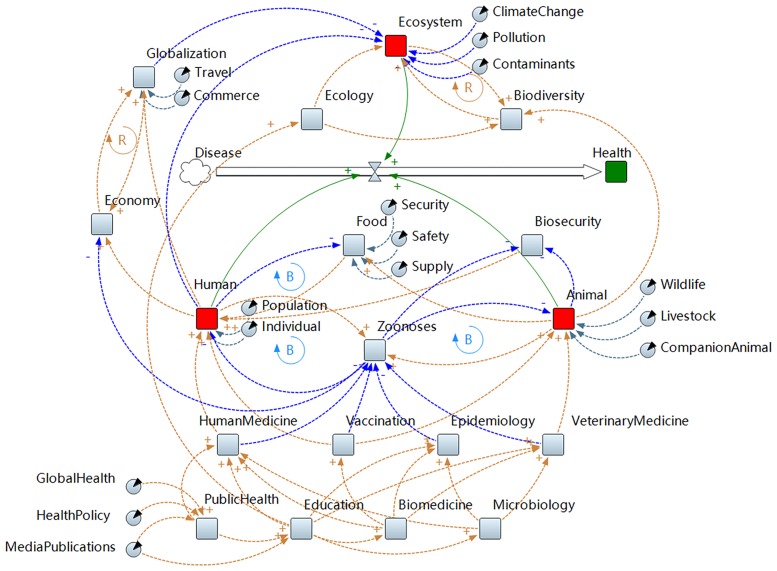
“One Health Cosmos”. One Health Cosmos shows the relationships between the various disciplines and complex problem descriptors that are reported to fall within the One Health concept. Squares and circles represent nodes, and the arrows connecting nodes represent causal links. Brown color is used to show positive causal link which also have a “+” sign besides the arrowhead. Negative causal link is portrayed with a blue color and “-” sign besides the arrowhead. A positive causal link means that both the causative and the resultant factors increase or decrease in the same direction. A negative causal link indicates that the two linked factors change in opposite directions. The positive reinforcing loop has a “R” in the clockwise cycle. A negative reinforcing loop opposite has a “B” in the counterclockwise cycle. A big arrow shows the direction of this relationship between disease and health through One Health.

In the first level of our model, we demonstrate the connection between disease with health using an arrow that shows the direction of this relationship. We then added the human, animal, and ecosystem domains (second level) to the model. These terms are represented by the red squares which are used to denote stock elements. Next, the third level elements were added, denoted by squares with grey color, which were also derived from the reviewed articles. Then, variable elements associated with each of the three One Health domains were added, denoted by circular shapes. These variable elements are assigned as descriptive components of the stocks. Last, causal links as previously described, were drawn between the elements. This model (we have learned the “One Health Cosmos”, Fig 2) was constructed using AnyLogic^®^ PLE software, version 7.3.4 (AnyLogic North America, LLC, Chicago, IL, USA).

### Model Evaluation (Step 4)

Our analysis focused upon peer-reviewed articles specifically discussing the One Health concept, which were selected as described by the search criteria. Some articles were excluded because they were not focused on the overall concept of One Health, just briefly mentioned the concept, or only covered a particular practice of One Health (exclusion criteria). While these papers may have been excluded, it is important to note that many of the specific topics they discuss, such as zoonoses and food safety, in the context of One Health practice, are still accounted for in our model. Thus, the model likely benefits from the removal of those reports so that the conceptual framework is centered on more specific interpretations of the One Health concept.

A term that has been used synonymously to One Health is comparative/translational medicine, which in fact was used before the One Health term was introduced [2, 3, 6, 25]. However, comparative/translational medicine was not used as a search term in this model given that the One Health concept has instead been more readily accepted as the more common term to use. Furthermore, compared to comparative/translational medicine, One Health has a broader application in scope [15].

## Discussion

From our analysis of the One Health literature and through the construction of the One Health Cosmos using a system dynamics theoretical model, we were able to derive multiple inferences.

Our results make it clear that One Health represents a wide-ranging synergistic field that is rapidly growing. This is reflected in the number of One Health articles that have been published in the past 25 years, which has seen an average publication increase of 14.6% per year during this time [23]. As a result, the interpretation of the One Health concept remains unclear because its internal relationships between this growing list of various components have not been systematically described.

Our model indicates that humans and animals are closely connected by zoonoses and the food supply chain [34, 35]. Some common zoonoses include avian influenza [36, 37], leishmaniasis [38], Middle East respiratory syndrome coronavirus prevention [39], rabies [40, 41] etc. Additionally, the rising demand for animal protein for human consumption, resulting in a shift towards larger, more complex animal production operations, has spurred the increased use of antimicrobials. In fact, experts estimate that from 2010 and 2030, the global consumption of antimicrobials will increase by 67% [42]. As such, investing more resources into the research and control of zoonotic diseases and complex food production systems could be an effective strategy for improving the overall health of humans and animals.

Our model also shows that zoonoses are closely associated with the economy, and can result in extensive economic losses when large zoonotic diseases outbreaks among humans and animals occur. For example, after the 2014 outbreak of Ebola virus, it was estimated that the affected countries of Guinea, Liberia, and Sierra Leone alone lost $2.2 billion in gross domestic product (GDP) the following year [43]. Additionally, the 2014 highly pathogenic avian influenza (HPAI) H5N2 outbreak in the United States, resulted in an estimated economic loss of $3.3 billion [44]. Hence, given the strength of association between zoonoses and the economy as demonstrate by the model, investing in prevention measures could result in an overall cost savings as new potential outbreaks are avoided [45].

Also, the health of companion animals is a growing concern. Pet keeping is a traditional custom for Western countries and becoming a new trend for non-Western countries. Pets could also bring disease to pet owners or non-pet owners who lives in close with them [46], such as echinococcosis, toxocarosis [47–49]. The One Health concept is required for a good understanding of the parasites’ biology and epidemiology and the education of pet owners.

We also found that education plays a foundational role in the development of the One Health concept. One Health education can be divided into education of those already working in the relevant professional disciplines, and of students seeking professional qualifications to enter one of these disciplines [2]. As One Health continues to be accepted as a way forward, there will be an increasing need to integrate the One Health concept into related disciplines’ education [50, 51]. Our model currently links education to epidemiology, human medicine, veterinary medicine, ecology, microbiology, and biomedicine. However, the depth of current education in One Health is unknown. Seems likely that education is not standardized across disciplines.

This report has a number of limitations. Despite our comprehensive approach we likely missed some important One Health publications in the literature review. Likewise, we may have failed to identify some important factors or components engaged in One Health activities.

To date, our analyses is the first to apply a system dynamics approach to understanding the One Health concept. Such an approach is useful in further elucidating the complex connections and causal factors that currently comprise One Health. By showing the inter-relationships between the various components of One Health, offering a visual example of the conceptual framework, the One Health Cosmos can help facilitate a more concise interpretation of the One Health concept for the development of future research and training programs.

## Supporting information

S1 File PRISMA 2009 checklist. (PDF)

## References

[1] Gibbs P. Origins of One Health and One Medicine. The Veterinary record. 2014;174(6):152 10.1136/vr.g1372. . 24509397

[2] Gibbs EPJ. The evolution of One Health: a decade of progress and challenges for the future. Veterinary Record. 2014;174(4):85–91. Epub 2014/01/28. 10.1136/vr.g143 . 24464377

[3] Atlas RM. One Health: Its Origins and Future One Health: The Human-Animal-Environment Interfaces in Emerging Infectious Diseases: The Concept and Examples of a One Health Approach. Current Topics in Microbiology and Immunology. 3652013. p. 1–13. 10.1007/82_2012_223 22527177

[4] Kahn LHMDMPHMPPF, Kaplan BDVM, Monath TPMD, Steele JHDVMMPH. Teaching "One Medicine, One Health". The American Journal of Medicine. 2008;121(3):169 10.1016/j.amjmed.2007.09.023 18328295 PMC7119384

[5] Taylor LH, Latham SM, Woolhouse ME. Risk factors for human disease emergence. Philosophical transactions of the Royal Society of London Series B, Biological sciences. 2001;356(1411):983–9. 10.1098/rstb.2001.0888 . 11516376 PMC1088493

[6] Evans BR, Leighton FA. A history of One Health. Revue scientifique et technique (International Office of Epizootics). 2014;33(2):413–20. 25707172 10.20506/rst.33.2.2298

[7] Gibbs SEJ, Gibbs EPJ. The historical, present, and future role of veterinarians in One Health. Current topics in microbiology and immunology. 2013;365:31–47. 10.1007/82_2012_259. 22911439 PMC7121980

[8] King LJ, Anderson LR, Blackmore CG, Blackwell MJ, Lautner EA, Marcus LC, et al Executive summary of the AVMA One Health Initiative Task Force report. Journal of the American Veterinary Medical Association. 2008;233(2):259–61. 10.2460/javma.233.2.259 . 18627228

[9] Gibbs EP, Anderson TC. One World—One Health' and the global challenge of epidemic diseases of viral aetiology. Veterinaria italiana. 2009;45(1):35–44. . 20391388

[10] Monath TP, Kahn LH, Kaplan B. Introduction: One Health Perspective. ILAR journal. 2010;51(3):193–8. 21131719 10.1093/ilar.51.3.193

[11] Coker R, Rushton J, Mounier-Jack S, Karimuribo E, Lutumba P, Kambarage D, et al Towards a conceptual framework to support one-health research for policy on emerging zoonoses. The Lancet infectious diseases. 2011;11(4):326–31. Epub 2011/03/08. 10.1016/S1473-3099(10)70312-1 . 21376670 PMC7129889

[12] Lee K, Brumme ZL. Operationalizing the One Health approach: the global governance challenges. Health Policy Plan. 2013;28(7):778–85. 10.1093/heapol/czs127 . 23221123

[13] Craddock S, Hinchliffe S. One world, one health? Social science engagements with the one health agenda. Social science & medicine (1982). 2015;129:1–4. 10.1016/j.socscimed.2014.11.016 . 25434985

[14] One-Health-Initiative. One Health Initiative—About One Health http://www.onehealthinitiative.com/about.php: One Health Initiative; 2010 [cited 2016 (Accessed 2016.08.20)]. http://www.onehealthinitiative.com/about.php.

[15] Lerner H, Berg C. The concept of health in One Health and some practical implications for research and education: what is One Health? Infection ecology & epidemiology. 2015;5:25300 Epub 2015/02/11. 10.3402/iee.v5.25300 . 25660757 PMC4320999

[16] Forrester JW. Industrial Dynamics—a Major Breakthrough for Decision Makers. Harvard Bus Rev. 1958;36(4):37–66.

[17] Royston G, Dost A, Townshend J, Turner H. Using system dynamics to help develop and implement policies and programmes in health care in England. System Dynamics Review. 1999;15(3):293–313. 10.1002/(Sici)1099-1727(199923)15:3<293::Aid-Sdr169>3.3.Co;2-T

[18] Ghaffarzadegan N, Lyneis J, Richardson GP. How small system dynamics models can help the public policy process. System Dynamics Review. 2011;27(1):22–44. 10.1002/sdr.442

[19] Günal MM, Pidd M. Discrete event simulation for performance modelling in health care: a review of the literature. Journal of Simulation. 2010;4(1):42–51.

[20] Homer JB, Hirsch GB. System dynamics modeling for public health: background and opportunities. Am J Public Health. 2006;96(3):452–8. 10.2105/AJPH.2005.062059 . 16449591 PMC1470525

[21] Homer J, Hirsch G, Minniti M, Pierson M. Models for collaboration: how system dynamics helped a community organize cost—effective care for chronic illness. System Dynamics Review. 2004;20(3):199–222.

[22] Hirsch G, Homer J, Evans E, Zielinski A. A system dynamics model for planning cardiovascular disease interventions. Am J Public Health. 2010;100(4):616–22. 10.2105/AJPH.2009.159434 . 20167899 PMC2836333

[23] Manlove KR, Walker JG, Craft ME, Huyvaert KP, Joseph MB, Miller RS, et al "One Health" or Three? Publication Silos Among the One Health Disciplines. Plos Biol. 2016;14(4). 10.1371/journal.pbio.1002448 27100532 PMC4839662

[24] Cardiff RD, Ward JM, Barthold SW. 'One medicine-one pathology': are veterinary and human pathology prepared? Laboratory Investigation. 2008;88(1):18–26. 10.1038/labinvest.3700695 18040269 PMC7099239

[25] Frank D. One world, one health, one medicine. Canadian Veterinary Journal-Revue Veterinaire Canadienne. 2008;49(11):1063–+. PMC2572090 19183729

[26] Zinsstag J, Schelling E, Waltner-Toews D, Tanner M. From "one medicine" to "one health" and systemic approaches to health and well-being. Preventive veterinary medicine. 2011;101(3–4):148–56. Epub 2010/09/14. 10.1016/j.prevetmed.2010.07.003 . 20832879 PMC3145159

[27] Rabozzi G, Bonizzi L, Crespi E, Somaruga C, Sokooti M, Tabibi R, et al Emerging zoonoses: the "one health approach". Safety and health at work. 2012;3(1):77–83. 10.5491/SHAW.2012.3.1.77 MEDLINE:22953235. 22953235 PMC3430925

[28] Zinsstag J, Mackenzie JS, Jeggo M, Heymann DL, Patz JA, Daszak P. Mainstreaming One Health. EcoHealth. 2012;9(2):107–10. 10.1007/s10393-012-0772-8. 22777051 PMC3415611

[29] Calistri P, Iannetti S, Danzetta ML, Narcisi V, Cito F, Di Sabatino D, et al The Components of 'One World—One Health' Approach. Transboundary and emerging diseases. 2013;60:4–13. 10.1111/tbed.12145 24589096

[30] Bidaisee S, Macpherson CNL. Zoonoses and One Health: A Review of the Literature. Journal of parasitology research. 2014:874345-. ZOOREC:ZOOR15010050282. 10.1155/2014/874345 24634782 PMC3928857

[31] Stephen C, Karesh WB. Is One Health delivering results? Introduction. Revue Scientifique Et Technique-Office International Des Epizooties. 2014;33(2):375–9. 10.20506/rst.33.2.2301 25707169

[32] Stadtlander CTKH. One Health: people, animals, and the environment. Infection ecology & epidemiology. 2015;5 10.3402/iee.v5.30514. PMC4698432 26725522

[33] Kingsley P, Taylor EM. One Health: competing perspectives in an emerging field. Parasitology. 2016:1–8. Epub 2016/01/29. 10.1017/s0031182015001845 . 26817944

[34] Rock M, Buntain BJ, Hatfield JM, Hallgrimsson B. Animal-human connections, "one health," and the syndemic approach to prevention. Social science & medicine (1982). 2009;68(6):991–5. Epub 2009/01/23. 10.1016/j.socscimed.2008.12.047 . 19157669

[35] Yates-Doerr E. The world in a box? Food security, edible insects, and "One World, One Health" collaboration. Social science & medicine (1982). 2015;129:106–12. 10.1016/j.socscimed.2014.06.020 . 24973999

[36] Capua I. Joining the dots on the emergence of pandemic influenza. Journal of clinical virology: the official publication of the Pan American Society for Clinical Virology. 2013;58(2):342–3. Epub 2013/05/07. 10.1016/j.jcv.2013.04.001 . 23643193

[37] Sims LD, Peiris M. One Health: The Hong Kong Experience with Avian Influenza. One Health: The Human-Animal-Environment Interfaces in Emerging Infectious Diseases: The Concept and Examples of a One Health Approach Current Topics in Microbiology and Immunology. 3652013. p. 281–98. 10.1007/82_2012_254 PMC7120750 22903569

[38] Palatnik-de-Sousa CB, Day MJ. One Health: the global challenge of epidemic and endemic leishmaniasis. Parasites & vectors. 2011;4:197 Epub 2011/10/12. 10.1186/1756-3305-4-197 . 21985335 PMC3214158

[39] Zumla A, Dar O, Kock R, Muturi M, Ntoumi F, Kaleebu P, et al Taking forward a 'One Health' approach for turning the tide against the Middle East respiratory syndrome coronavirus and other zoonotic pathogens with epidemic potential. International journal of infectious diseases: IJID: official publication of the International Society for Infectious Diseases. 2016 Epub 2016/06/21. 10.1016/j.ijid.2016.06.012 . 27321961 PMC7128966

[40] Manoj S, Mukherjee A, Johri S, Kumar KV. Recovery from rabies, a universally fatal disease. Mil Med Res. 2016;3:21 10.1186/s40779-016-0089-y ; 27429788 PMC4947331

[41] Perez de Diego AC, Vigo M, Monsalve J, Escudero A. The One Health approach for the management of an imported case of rabies in mainland Spain in 2013. Euro surveillance: bulletin Europeen sur les maladies transmissibles = European communicable disease bulletin. 2015;20(6). Epub 2015/02/20. . 25695478 10.2807/1560-7917.es2015.20.6.21033

[42] Van Boeckel TP, Brower C, Gilbert M, Grenfell BT, Levin SA, Robinson TP, et al Global trends in antimicrobial use in food animals. Proc Natl Acad Sci U S A. 2015;112(18):5649–54. 10.1073/pnas.1503141112 ; 25792457 PMC4426470

[43] CDC. Cost of the Ebola Epidemic Centers for Disease Control and Prevention: Centers for Disease Control and Prevention; 2016 [updated August 8, 2016; cited 2016 (Accessed 2016.08.25)]. http://www.cdc.gov/vhf/ebola/outbreaks/2014-west-africa/cost-of-ebola.html.

[44] Greene JL. Update on the Highly-Pathogenic Avian Influenza Outbreak of 2014–2015. Congressional Research Service. 2015:1–18.

[45] Rushton J, Häsler B, De Haan N, Rushton R. Economic benefits or drivers of a'One Health'approach: Why should anyone invest? Onderstepoort Journal of Veterinary Research. 2012;79(2):75–9. 10.4102/ojvr.v79i2.461 23327381

[46] Rock MJ, Adams CL, Degeling C, Massolo A, McCormack GR. Policies on pets for healthy cities: a conceptual framework. Health promotion international. 2015;30(4):976–86. Epub 2014/04/04. 10.1093/heapro/dau017 . 24694682 PMC4651052

[47] Deplazes P, van Knapen F, Schweiger A, Overgaauw PAM. Role of pet dogs and cats in the transmission of helminthic zoonoses in Europe, with a focus on echinococcosis and toxocarosis. Veterinary parasitology. 2011;182(1):41–53. 10.1016/j.vetpar.2011.07.014 21813243

[48] Dantas-Torres F, Otranto D. Dogs, cats, parasites, and humans in Brazil: opening the black box. Parasites & vectors. 2014;7:22 Epub 2014/01/16. 10.1186/1756-3305-7-22 . 24423244 PMC3914713

[49] Maia C, Ramos C, Coimbra M, Bastos F, Martins A, Pinto P, et al Bacterial and protozoal agents of feline vector-borne diseases in domestic and stray cats from southern Portugal. Parasites & vectors. 2014;7 10.1186/1756-3305-7-115 24655431 PMC3972989

[50] Lane SL. Integrated environmental education: Introducing One Health concepts into veterinary technician education [1590047]. Ann Arbor: Montreat College; 2015.

[51] Barrett MA, Bouley TA, Stoertz AH, Stoertz RW. Integrating a One Health approach in education to address global health and sustainabilrty challenges. Frontiers in Ecology and the Environment. 2011;9(4):239–45. 10.1890/090159.

